# Overwintering of Usutu virus in mosquitoes, The Netherlands

**DOI:** 10.1186/s13071-024-06620-y

**Published:** 2024-12-23

**Authors:** C. J. M. Koenraadt, E. Münger, M. J. J. Schrama, J. Spitzen, S. Altundag, R. S. Sikkema, B. B. Oude Munnink, M. P. G. Koopmans, R. Blom

**Affiliations:** 1 Laboratory of Entomology, Plant Sciences Group, Wageningen University, Wageningen, The Netherlands; 2 Viroscience, Erasmus MC, Rotterdam, The Netherlands; 3 Institute of Environmental Sciences, Leiden University, Leiden, The Netherlands; 4 Netherlands Institute for Vectors, Invasive Plants and Plant Health (NIVIP), Centre for Monitoring of Vectors (CMV), Netherlands Food and Consumer Product Safety Authority (NVWA), Wageningen, The Netherlands; 5 Centre for Avian Migration, Netherlands Institute of Ecology (NIOO-KNAW), Wageningen, The Netherlands

## Abstract

Analyses of mosquito-borne virus outbreaks have revealed the presence of similar virus strains over several years. However, it remains unclear how mosquito-borne viruses can persist over winter, when conditions are generally unfavorable for virus circulation. One potential route for virus persistence is via diapausing mosquitoes. We therefore studied whether Usutu virus (USUV), West Nile virus (WNV) and/or Sindbis virus (SINV) can be identified in diapausing mosquitoes in The Netherlands. Mosquito collections were carried out in November 2022 in hibernacula located in two areas with previously observed WNV and/or USUV activity. A total of 4857 mosquitoes, belonging to four species (groups) (*Culex pipiens/torrentium*, *Culiseta annulata*, *Anopheles maculipennis* s.l. and *Culex territans*), were collected. WNV-, USUV- and SINV-screening using a multiplex real-time RT-PCR assay was carried out on mono-specific mosquito pools. One *Culex pipiens*/*torrentium* pool tested positive for USUV RNA. Whole genome sequencing and subsequent phylogenetic analysis revealed that the virus belongs to USUV lineage Africa 3 and clusters with other USUV sequences derived from The Netherlands in 2022. This finding confirms our hypothesis of the potential of local overwintering of USUV in diapausing mosquitoes in The Netherlands.

**Graphical Abstract:**

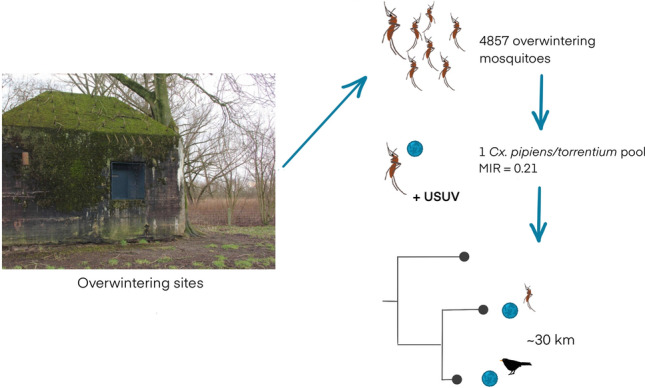

## Background

In recent years, Europe has witnessed increased circulation of mosquito-borne viruses that have an impact on human and animal health. These include West Nile (WNV) and Usutu (USUV) virus (*Orthoflavivirus*, *Flaviviridae*), and there is growing concern that climate conditions are becoming more conducive for virus transmission [1, 2]. Recurring outbreaks of arboviruses within a specific region raise questions about the extent to which these arboviruses can locally overwinter, or whether they are introduced annually, followed by continued spread of the virus. In case of repeated introductions, different (migratory) bird species may carry the virus from areas where the virus actively circulates to areas where it does not yet circulate but where it can be picked up by locally competent vector species [3]. The dynamics of these events are strongly driven by ecological traits of birds, including their flight distance, migratory patterns (including stop-over) and susceptibility to the virus. In case of local circulation across years, the virus may be sustained in either a vector or a host species during unfavorable winter conditions. When temperatures become more favorable in spring, virus transmission may then resume. Which of the two scenarios occurs will be relevant for assessing transmission risk and will direct surveillance efforts regarding vectors, hosts and viruses.

The main vector of USUV and WNV in Europe is *Culex pipiens*. The species consists of two biotypes, *Cx. pipiens* biotype *pipiens* and *Cx. pipiens* biotype *molestus*, which have different host feeding preferences and therefore play a different role in arbovirus transmission [4, 5]. The ornithophilic *pipiens* biotype is likely to be the main enzootic vector of both arboviruses, transmitting the virus among bird populations. Within The Netherlands, biotype *pipiens* is known to bite a broad range of bird species, ranging from small passerines such as the European Robin (*Erithacus rubecula*) and Eurasian Blue Tit (*Cyanistes cauruleus*) to wading birds, e.g. Gray Heron (*Ardea cinerea*) and domestic chickens (*Gallus gallus*), while the *molestus* biotype mainly bites humans and other mammal species [6]. Besides host preference, the two biotypes markedly differ in their overwintering strategy, with the *molestus* biotype remaining active in winter [7]. In contrast, the *pipiens* biotype enters a state of reduced metabolic activity, arrested ovarian development and increased fat body reserves in winter, known as diapause [8–10]. This diapause is induced in the larval stages by lower temperatures (< 10 °C) and reduced daylength in autumn. Emerging adult mosquitoes will then feed on sugars to build up their fat reserves and will no longer blood feed. From around October onwards, they will seek shelter in animal burrows, cellars and other generally dark and climatically stable places to survive adverse weather conditions [11–14]. It has been estimated that up to 70% of diapausing *Cx. pipiens* may survive winter, and those surviving will initiate biting when climate conditions become favorable the following spring (in The Netherlands this is around 4 to 6 months after start of diapause) [13]. If infected, this may also resume virus circulation.

Detecting virus in the mosquito population is notoriously difficult, as virus infection rates are usually very low [15]. Therefore, little is known about virus infection rates over the seasons, including what happens when mosquitos enter diapause. Earlier work on genomic sequences from bird-derived USUV demonstrated that annual re-occurrence of related strains takes place, suggesting that it likely overwinters in The Netherlands [16]. Across Europe, USUV screening of diapausing mosquitoes has only been performed sporadically; thus far, USUV evidence in overwintering mosquitoes is limited to an anecdotal detection in Austria and a single USUV-positive pool of *Culex torrentium* in Poland [17, 18]. The first findings of WNV RNA in diapausing mosquitoes in Europe were reported in 2017, when WNV RNA was detected in three pools (out of 573) of diapausing *Culex pipiens* mosquitoes collected in February or March in the Czech Republic [19]. Additional WNV-positive pools were found in diapausing *Cx. pipiens* mosquitoes from the same area several years later [20]. Also in Germany, evidence of WNV overwintering in diapausing *Cx. pipiens* mosquitoes was found in the winter of 2020/2021 [21]. Furthermore, Sindbis virus (SINV; *Alphavirus, Togaviridae*) has been observed in diapausing mosquitoes in Sweden [22]. Presence of this virus in mosquitoes in The Netherlands is also suspected based on recent findings of SINV in birds and horses [23, 24] (Streng and Holicki et al. under review).

Mosquitoes that enter diapause as adults will no longer blood feed during the subsequent winter. Therefore, if an arbovirus overwinters in mosquitoes, it needs to have been vertically transmitted from mother to her offspring. Only in this way do larvae become infected, and may emerge as infected adults. As vertical transmission is thought to be a rare event and as virus infection rates are very low in general, it is even more challenging to detect arboviruses in overwintering mosquito populations than in summer populations [25–27]. Our previous report concluded that there was no evidence of arbovirus overwintering in mosquito populations from The Netherlands [11]. Here, we report the first case of an USUV-infected pool of diapausing mosquitoes and further describe its phylogenetic relationship with known circulating virus strains based on the virus's genetic sequence.

## Methods

### Mosquito collection and virus detection

Mosquito collection, identification and sample processing were done according to protocols described in our earlier work [11]. In brief, mosquito collections were carried out once in November 2022 in the municipalities of Stichtse Vecht and Utrecht (area A) and West-Betuwe (area B), The Netherlands. Human-made structures, including (bat) cellars, wells, chicken pens and late nineteenth-century bunkers of the New Hollandic Waterline were sampled. These sites are known to host a range of mosquito species as well as other insects and bats [11, 13, 28]. Manual and/or automatic aspiration was used to collect mosquitoes. Morphological mosquito identification was performed using the identification key described by Becker et al. [29]. Monospecific mosquito pools with a maximum of ten mosquitoes per pool were made in medium. Detection of USUV, WNV and SINV was done on these samples by multiplex real-time RT-PCR as previously described [11]. The USUV positive result was confirmed by a second RT-PCR [30].

### Viral whole genome sequencing and sequence data analysis

The single USUV RT-PCR-positive RNA sample was submitted to whole genome sequencing using an amplicon-based approach on Oxford Nanopore technology as previously described [16, 31]. In short, random primers (Invitrogen) were used to perform reverse transcription using ProtoScript II (NEB, cat. No. E6569) after which USUV-specific multiplex PCR was performed in two reactions using Q5 Hot Start High-Fidelity DNA Polymerase (NEB, cat no. M0493). Nanopore sequencing was performed according to the manufacturer’s instructions using the Ligation sequencing kit (SQK-LSK110) with Native Barcoding Kit 96 V14 (SQK-NBD114.96) on a FLO-MIN106D R9.4.1 Flow Cell (Oxford Nanopore Technologies). Raw sequence data were demultiplexed, and reads were quality controlled to a minimum length of 150 and a median PHRED score of 10 using FastP [32]. A reference-based alignment was performed using Minimap2 [33]. A consensus genome was extracted, reads were remapped to this consensus sequence, and a new consensus sequence was generated. Positions with < 30 coverage were replaced with an ‘N’. Homopolymeric and primer binding regions were manually checked and resolved by consulting reference genomes.

### Phylogenetic analysis

All complete USUV genome sequences (length > 8800 bp) were retrieved from GenBank (https://doi.org/10.1093/nar/gkp1024) on May 2024. The reference sequences were aligned with unpublished USUV genome sequences obtained from birds and mosquitoes in The Netherlands between 2016 and 2022 [34] as well as with the USUV genome from the diapausing mosquito pool generated in this study using MAFFT version 7.475 [35]. The alignment was manually checked for discrepancies. A maximum likelihood phylogenetic tree was estimated using IQ-TREE version 2.0.3 [36] under a GTR + F + I + G4 model. Branch supports were assessed using ultrafast bootstrap approximation [37] with 1000 bootstrap replicates. The resulting phylogenetic tree was midpoint rooted.

## Results

In November 2022, a total number of 534 mosquitoes (61 pools) was collected from area A: 528 *Cx. pipiens/torrentium* (98.9%), 2 *Culiseta annulata* (0.4%), 3 *Anopheles maculipennis* s.l. (0.6%) and 1 *Culex territans* (0.2%). From area B, a total number of 4323 mosquitoes (443 pools) was collected in November 2022; 3416 *Cx. pipiens/torrentium* (79.0%), 320 *Cs. annulata* (7.4%), 482 *An. maculipennis* s.l. (11.1%) and 105 *Cx. territans* (2.4%). Arbovirus screening resulted in the detection of one USUV-positive pool (Ct value = 23.68) consisting of ten *Cx. pipiens/torrentium* mosquitoes collected from a bunker in area B in November 2022. All other 503 pools tested negative for USUV, WNV and SINV.

**Fig. 1:**
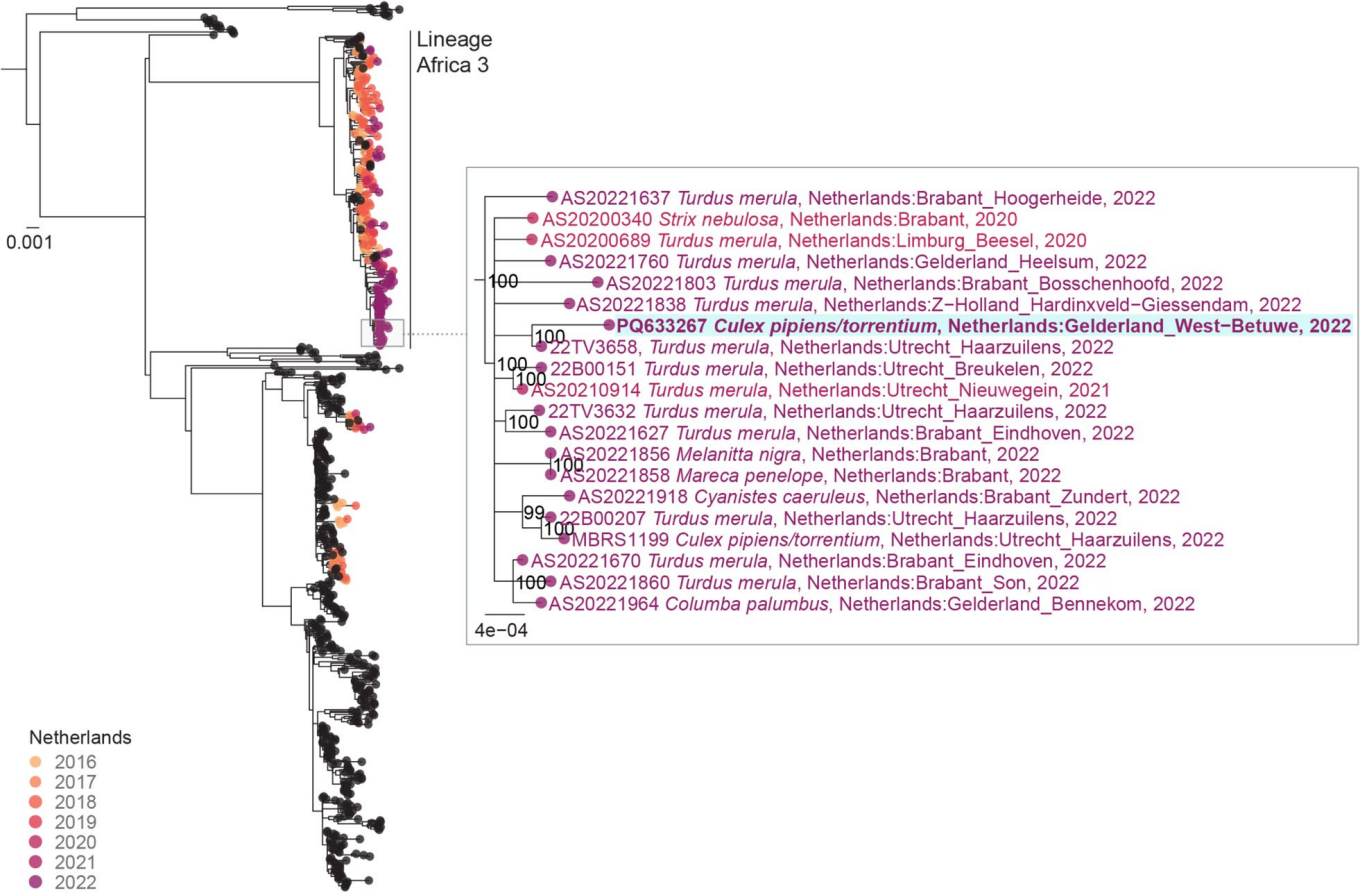
Maximum likelihood phylogeny of USUV genome sequences, with tips corresponding to sequences from The Netherlands colored by year of sampling (yellow for 2016 to purple for 2022). A detailed view shows the position of the sequence obtained from a pool of diapausing *Cx. pipiens/torrentium* mosquitoes highlighted in blue. In the zoomed-in section, labels include host species, location and year of collection; bootstrap values > 80 are indicated next to the nodes. Scale units are nucleotide substitutions per site

A near full-length USUV genome sequence was obtained from the USUV-positive *Cx. pipiens/torrentium* pool. Phylogenetic analysis showed that the virus identified in the diapausing mosquitoes belonged to the USUV lineage Africa 3 (Fig. 1) and was most closely related to a sequence obtained from a live blackbird (*Turdus merula*) collected from De Haar in the same year (30 km away from the mosquito sampling location).

## Discussion

In this study, we aimed to assess whether mosquito-borne viruses, which caused disease outbreaks among birds and humans during summer and autumn in The Netherlands, can overwinter in diapausing mosquitoes. One *Cx. pipiens/torrentium* pool tested positive for USUV lineage Africa 3, whereas other mosquito pools all tested negative for WNV, USUV or SINV. Lineage Africa 3 is the most frequently detected USUV lineage in The Netherlands. Known to circulate in the country since 2016 [16], it was also detected in birds and mosquitoes in summer and autumn of 2022, including in the region of Utrecht [34]. Annual re-emergence of related strains within this lineage suggests local persistence and therewith establishment of the virus in the region. Our finding shows that USUV lineage 3 can persist in diapausing mosquitoes, which can be one pathway for this virus to overwinter.

In our study, the minimum infection rate (MIRs [= number of positive pools/total number of tested specimens*1000]) for USUV in diapausing mosquitoes in 2022 was 0.21. In Poland, researchers found a similar MIR of 0.18 for USUV in *Cx. torrentium* mosquitoes [18]. No other USUV MIRs have been reported. Low MIRs can either reflect actual infection rates or be the consequence of a low detectability of viruses. Earlier studies have shown that under winter conditions, virus replication in mosquitoes is low but may increase at higher temperatures [38, 39]. Consequently, there is a possibility that a fraction of the mosquitoes tested in our study were infected, but titers remained below the detection limit.

Arbovirus persistence may be driven by other potential overwintering routes, although the evidence remains scarce. In general, the role of other mosquito vectors (e.g. *Culex modestus*), non-mosquito vectors (e.g. ticks feeding on birds), non-avian reservoir hosts (such as mammals, reptiles and amphibians) as well as long-term viral persistence in bird hosts remains mostly unexplored, and additional research may provide new insights in arbovirus overwintering mechanisms [3, 40, 41]. Interestingly, WNV has been identified recently in winter-active *Cx. pipiens* in Greece [42]. Winter temperatures in Greece are, however, more favorable than in The Netherlands for continued mosquito activity. The lack of diapause may result in year-round transmission, which is not the case in The Netherlands with the current climate in winter.

In conclusion, we show that USUV can persist in diapausing *Cx. pipiens/torrentium* mosquitoes, albeit at low frequency. For WNV, no evidence has been found of local overwintering in diapausing mosquitoes in The Netherlands. Furthermore, we show that anthropogenic overwintering sites, such as bunkers, provide shelter for arbovirus-infected mosquitoes. To improve predictions of mosquito-borne disease outbreaks and to understand the role of mosquitoes in transmitting pathogens from animals to humans, it remains necessary to explore other potential routes of arbovirus persistence while also continuing research on arbovirus overwintering in mosquitoes.

## Data Availability

The genomic sequence of Usutu virus generated in this study has been deposited in the GenBank database under accession no. PQ633267.

## References

[1] Bakonyi T, Haussig JM. West Nile virus keeps on moving up in Europe. Eurosurveillance. 2020;25:pii=001938. 10.2807/1560-7917.ES.2020.25.46.2001938. 10.2807/1560-7917.ES.2020.25.46.2001938 PMC7678036 33213684

[2] Vilibic-Cavlek T, Petrovic T, Savic V, Barbic L, Tabain I, Stevanovic V, et al. Epidemiology of Usutu virus: the european scenario. Pathogens. 2020;9:699. 10.3390/pathogens9090699. 32858963 10.3390/pathogens9090699 PMC7560012

[3] Reisen WK, Wheeler SS. Overwintering of west nile virus in the United States. J Med Entomol. 2019;56:1498–507. 10.1093/jme/tjz070. 31549726 10.1093/jme/tjz070

[4] Brugman VA, Hernández-Triana LM, Medlock JM, Fooks AR, Carpenter S, Johnson N. The role of Culex pipiens L. (Diptera: Culicidae) in virus transmission in Europe. Int J Environ Res Public Health. 2018;15:389. 10.3390/ijerph15020389. 29473903 10.3390/ijerph15020389 PMC5858458

[5] ECDC. *Culex pipiens*—Factsheet for experts 2020. https://www.ecdc.europa.eu/en/infectious-disease-topics/related-public-health-topics/disease-vectors/facts/mosquito-factsheets/culex-pipiens. Accessed 19 Sep 2023.

[6] Blom R, Krol L, Langezaal M, Schrama M, Trimbos KB, Wassenaar D, et al. Blood-feeding patterns of *Culex pipiens* biotype *pipiens* and *pipiens/molestus* hybrids in relation to avian community composition in urban habitats. Parasites Vectors. 2024;17:95. 10.1186/s13071-024-06186-9. 38424573 10.1186/s13071-024-06186-9 PMC10902945

[7] Vogels CBF, Van De Peppel LJJ, Van Vliet AJH, Westenberg M, Ibañez-Justicia A, Stroo A, et al. Winter activity and aboveground hybridization between the two biotypes of the West Nile Virus vector *Culex pipiens*. Vector-Borne Zoonotic Dis. 2015;15:619–26. 10.1089/vbz.2015.1820. 26394124 10.1089/vbz.2015.1820

[8] Robich RM, Denlinger DL. Diapause in the mosquito *Culex pipiens* evokes a metabolic switch from blood feeding to sugar gluttony. Proc Natl Acad Sci USA. 2005;102:15912–7. 10.1073/pnas.0507958102. 16247003 10.1073/pnas.0507958102 PMC1276097

[9] Sim C, Denlinger DL. Insulin signaling and FOXO regulate the overwintering diapause of the mosquito *Culex pipiens*. Proc Natl Acad Sci. 2008;105:6777–81. 10.1073/pnas.0802067105. 18448677 10.1073/pnas.0802067105 PMC2373331

[10] Sim C, Denlinger DL. A shut-down in expression of an insulin-like peptide, ILP-1, halts ovarian maturation during the overwintering diapause of the mosquito *Culex pipiens*. Insect Mol Biol. 2009;18:325–32. 10.1111/j.1365-2583.2009.00872.x. 19523064 10.1111/j.1365-2583.2009.00872.x PMC3835429

[11] Blom R, Schrama MJJ, Spitzen J, Weller BFM, Van Der Linden A, Sikkema RS, et al. Arbovirus persistence in North-Western Europe: are mosquitoes the only overwintering pathway? One Health. 2023;16:100467. 10.1016/j.onehlt.2022.100467. 36531660 10.1016/j.onehlt.2022.100467 PMC9747676

[12] Dörge DD, Cunze S, Schleifenbaum H, Zaenker S, Klimpel S. An investigation of hibernating members from the *Culex pipiens* complex (Diptera, Culicidae) in subterranean habitats of central Germany. Sci Rep. 2020;10:10276. 10.1038/s41598-020-67422-7. 32581278 10.1038/s41598-020-67422-7 PMC7314823

[13] Koenraadt CJM, Möhlmann TWR, Verhulst NO, Spitzen J, Vogels CBF. Effect of overwintering on survival and vector competence of the West Nile virus vector *Culex pipiens*. Parasites Vectors. 2019;12:147. 10.1186/s13071-019-3400-4. 30917854 10.1186/s13071-019-3400-4 PMC6437999

[14] Sauer FG, Timmermann E, Lange U, Lühken R, Kiel E. Effects of hibernation site, temperature, and humidity on the abundance and survival of overwintering *Culex pipiens pipiens* and *Anopheles messeae* (Diptera: Culicidae). J Med Entomol. 2022;59:2013–21. 10.1093/jme/tjac139. 36130183 10.1093/jme/tjac139 PMC9667720

[15] Gu W, Lampman R, Novak RJ. Assessment of arbovirus vector infection rates using variable size pooling. Med Vet Entomol. 2004;18:200–4. 10.1111/j.0269-283X.2004.00482.x. 15189246 10.1111/j.0269-283X.2004.00482.x

[16] Oude Munnink BB, Münger E, Nieuwenhuijse DF, Kohl R, Linden AVD. Genomic monitoring to understand the emergence and spread of Usutu virus in the Netherlands, 2016–2018. Sci Rep. 2020;10:2798. 10.1038/s41598-020-59692-y. 32071379 10.1038/s41598-020-59692-y PMC7029044

[17] Pfeffer M, Dobler G. Emergence of zoonotic arboviruses by animal trade and migration. Parasit Vectors. 2010;3:35. 10.1186/1756-3305-3-35. 20377873 10.1186/1756-3305-3-35 PMC2868497

[18] Sauer FG, Lange U, Schmidt-Chanasit J, Kiel E, Wiatrowska B, Myczko Ł, et al. Overwintering *Culex torrentium* in abandoned animal burrows as a reservoir for arboviruses in Central Europe. One Health. 2023;16:100572. 10.1016/j.onehlt.2023.100572. 37363228 10.1016/j.onehlt.2023.100572 PMC10288133

[19] Rudolf I, Betá L, Bla H, Venclíková K, Straková P, Mendel J, et al. West Nile virus in overwintering mosquitoes, central Europe. Parasit Vectors. 2017;10:452. 10.1186/s13071-017-2399-7. 28969685 10.1186/s13071-017-2399-7 PMC5625652

[20] Rudolf I, Sikutova S, Sebesta O, Mendel J, Malenovsky I, Kampen H, et al. Overwintering of *Culex modestus* and other mosquito species in a reedbed ecosystem, including arbovirus findings. J Am Mosq Control Assoc. 2020;36:257–60. 10.2987/20-6949.1. 33647121 10.2987/20-6949.1

[21] Kampen H, Tews BA, Werner D. First evidence of west nile virus overwintering in mosquitoes in Germany. Viruses. 2021;13:2463. 10.3390/v13122463. 34960732 10.3390/v13122463 PMC8703620

[22] Bergman A, Dahl E, Lundkvist Å, Hesson JC. Sindbis virus infection in non-blood-fed hibernating *Culex pipiens* Mosquitoes in Sweden. Viruses. 2020;12:1441. 10.3390/v12121441. 33327649 10.3390/v12121441 PMC7765111

[23] Ziegler U, Fischer D, Eiden M, Reuschel M, Rinder M, Müller K, et al. Sindbis virus- a wild bird associated zoonotic arbovirus circulates in Germany. Vet Microbiol. 2019;239:108453. 10.1016/j.vetmic.2019.108453. 31767092 10.1016/j.vetmic.2019.108453

[24] ECDC. Facts about Sindbis fever 2023. https://www.ecdc.europa.eu/en/sindbis-fever/facts. Accessed 25 Nov 2024.

[25] Anderson JF, Main AJ, Delroux K. Extrinsic incubation periods for horizontal and vertical transmission of West Nile Virus by *Culex pipiens pipiens* (Diptera: Culicidae). J Med Entomol. 2008;45:455–451. 10.1603/0022-2585(2008)45[445:eipfha]2.0.co;2 18533438

[26] Anderson JF, Main AJ, Cheng G, Ferrandino FJ, Fikrig E. Horizontal and vertical transmission of West Nile Virus genotype NY99 by *Culex salinarius* and Genotypes NY99 and WN02 by *Culex tarsalis*. Am J Trop Med Hyg. 2012;86:134–9. 10.4269/ajtmh.2012.11-0473. 22232464 10.4269/ajtmh.2012.11-0473 PMC3247122

[27] Dohm DJ, Sardelis MR, Turell MJ. Experimental vertical transmission of West Nile Virus by *Culex pipiens* (Diptera: Culicidae). J Med Entomol. 2002;39:640–4. 10.1603/0022-2585-39.4.640. 12144296 10.1603/0022-2585-39.4.640

[28] Ibáñez-Justicia A, Smitz N, Blom R, Vanderheyden A, Jacobs F, Meganck K, et al. *Anopheles maculipennis* complex in The Netherlands: first record of *Anopheles daciae* (Diptera: Culicidae). Diversity. 2022;14:636. 10.3390/d14080636.

[29] Becker N, Petrić D, Zgomba M, Boase C, Madon MB, Dahl C, et al. Mosquitoes: identification, ecology and control. 3rd ed. Berlin, Heidelberg: Springer; 2020. 570p. 10.1007/978-3-030-11623-1.

[30] Jöst H, Bialonski A, Maus D, Sambri V, Eiden M, Groschup MH, et al. Isolation of Usutu Virus in Germany. Am Soc Trop Med Hyg. 2011;85:551–3. 10.4269/ajtmh.2011.11-0248. 10.4269/ajtmh.2011.11-0248 PMC3163883 21896821

[31] Oude Munnink BB, Kik M, De Bruijn ND, Kohl R, Van Der Linden A, Reusken CBEM, et al. Towards high quality real-time whole genome sequencing during outbreaks using Usutu virus as example. Infect Genet Evol. 2019;73:49–54. 10.1016/j.meegid.2019.04.015. 31014969 10.1016/j.meegid.2019.04.015

[32] Chen S, Zhou Y, Chen Y, Gu J. fastp: an ultra-fast all-in-one FASTQ preprocessor. Bioinformatics. 2018;34:i884–90. 10.1093/bioinformatics/bty560. 30423086 10.1093/bioinformatics/bty560 PMC6129281

[33] Li H. Minimap2: pairwise alignment for nucleotide sequences. Bioinformatics. 2018;34:3094–100. 10.1093/bioinformatics/bty191. 29750242 10.1093/bioinformatics/bty191 PMC6137996

[34] Münger E, et al. Emergence and dynamics of Usutu and West Nile viruses in the Netherlands, 2016–2022. https://www.biorxiv.org/content/10.1101/2024.12.16.628479v1. 10.1038/s41467-025-63122-w PMC12375057 40849294

[35] Katoh K, Standley DM. MAFFT multiple sequence alignment software version 7: improvements in performance and usability. Mol Biol Evol. 2013;30:772–80. 10.1093/molbev/mst010. 23329690 10.1093/molbev/mst010 PMC3603318

[36] Minh BQ, Schmidt HA, Chernomor O, Schrempf D, Woodhams MD, Von Haeseler A, et al. IQ-TREE 2: new models and efficient methods for phylogenetic inference in the genomic era. Mol Biol Evol. 2020;37:1530–4. 10.1093/molbev/msaa015. 32011700 10.1093/molbev/msaa015 PMC7182206

[37] Hoang DT, Chernomor O, Von Haeseler A, Minh BQ, Vinh LS. UFBoot2: improving the ultrafast bootstrap approximation. Mol Biol Evol. 2018;35:518–22. 10.1093/molbev/msx281. 29077904 10.1093/molbev/msx281 PMC5850222

[38] Dohm DJ, Turell MJ. Effect of incubation at overwintering temperatures on the replication of West Nile Virus in New York *Culex pipiens* (Diptera: Culicidae). J Med Entomol. 2001;38:462–4. 10.1603/0022-2585-38.3.462. 11372976 10.1603/0022-2585-38.3.462

[39] Jiang S, Xing D, Li C, Dong Y, Zhao T, Guo X. Replication and transmission of West Nile virus in simulated overwintering adults of *Culex pipiens pallens* (Diptera: Culicidae) in China. Acta Trop. 2023;237:106720. 10.1016/j.actatropica.2022.106720. 36288768 10.1016/j.actatropica.2022.106720

[40] Nemeth N, Young G, Ndaluka C, Bielefeldt-Ohmann H, Komar N, Bowen R. Persistent West Nile virus infection in the house sparrow (*Passer domesticus*). Arch Virol. 2009;154:783–9. 10.1007/s00705-009-0369-x. 19347246 10.1007/s00705-009-0369-x

[41] Kuno G. Persistence of arboviruses and antiviral antibodies in vertebrate hosts: its occurrence and impacts†. Rev Med Virol. 2001;11:165–90. 10.1002/rmv.314. 11376480 10.1002/rmv.314

[42] Balatsos G, Beleri S, Tegos N, Bisia M, Karras V, Zavitsanou E, et al. Overwintering West Nile virus in active *Culex pipiens* mosquito populations in Greece. Parasites Vectors. 2024;17:286. 10.1186/s13071-024-06367-6. 38956733 10.1186/s13071-024-06367-6 PMC11221078

